# Short‐term lung function changes predict mortality in patients with fibrotic hypersensitivity pneumonitis

**DOI:** 10.1111/resp.14204

**Published:** 2022-01-12

**Authors:** Claudio Macaluso, Cristina Boccabella, Maria Kokosi, Nishanth Sivarasan, Vasilis Kouranos, Peter M. George, George Margaritopoulos, Philip L. Molyneaux, Felix Chua, Toby M. Maher, Gisli R. Jenkins, Andrew G. Nicholson, Sujal R. Desai, Anand Devaraj, Athol U. Wells, Elisabetta A. Renzoni, Carmel J. W. Stock

**Affiliations:** ^1^ Interstitial Lung Disease Unit Royal Brompton and Harefield Clinical Group, Guy's and St Thomas' NHS Foundation Trust London UK; ^2^ Department of Pneumology INRCA/IRCCS, "L.Mandic" Hospital Merate (LC) Merate Italy; ^3^ Department of Medical and Surgical Sciences Fondazione Policlinico Universitario "A. Gemelli" – IRCCS, University of the Sacred Heart Rome Italy; ^4^ Department of Radiology Royal Brompton and Harefield Clinical Group, Guy's and St Thomas' NHS Foundation Trust London UK; ^5^ Margaret Turner Warwick Centre for Fibrosing Lung Disease National Heart and Lung Institute, Imperial College London London UK; ^6^ ILD Unit London North West University Hospital Healthcare Trust London UK; ^7^ Faculty of Biology, Medicine and Health The University of Manchester Manchester UK; ^8^ Hastings Centre for Pulmonary Research and Division of Pulmonary, Critical Care and Sleep Medicine Keck School of Medicine, University of Southern California Los Angeles California USA; ^9^ Department of Histopathology Royal Brompton and Harefield Clinical Group, Guy's and St Thomas' NHS Foundation Trust London UK

**Keywords:** DLCO, fibrotic hypersensitivity pneumonitis, FVC, mortality, predictor, short‐term lung function change

## Abstract

**Background and objective:**

A proportion of patients with fibrotic hypersensitivity pneumonitis (fHP) follow a progressive disease course despite immunosuppressive treatment. Little is known about predictors of mortality in fHP. We aimed to investigate the impact of short‐term lung function changes in fHP on mortality.

**Methods:**

Baseline demographics for 145 consecutive patients with a multi‐disciplinary team diagnosis of fHP, as well as baseline and 1‐year follow‐up of lung function, baseline echocardiographic findings, bronchoalveolar lavage (BAL) cellularity and all‐cause mortality were recorded. Changes in forced vital capacity (FVC) ≥ 5% and ≥10%, and diffusion capacity of the lung for carbon monoxide (DLCO) ≥ 10% and ≥15% at 1 year were calculated. Cox proportional hazards analysis was performed to test for associations with mortality.

**Results:**

Baseline lung function severity, age, presence of honeycombing on computed tomography (CT) and echocardiographic pulmonary arterial systolic pressure (PASP) ≥ 40 mm Hg were associated with early mortality, while BAL lymphocytosis was associated with improved survival. A decline in FVC ≥ 5% (hazard ratio [HR]: 3.10, 95% CI: 2.00–4.81, *p* < 0.001), FVC ≥ 10% (HR: 3.11, 95% CI: 1.94–4.99, *p* < 0.001), DLCO ≥ 10% (HR: 2.80, 95% CI: 1.78–4.42, *p* < 0.001) and DLCO ≥ 15% (HR: 2.92, 95% CI: 1.18–4.72, *p* < 0.001) at 1 year was associated with markedly reduced survival on univariable and multivariable analyses after correcting for demographic variables, disease severity, honeycombing on CT and treatment, as well as BAL lymphocytosis and PASP ≥ 40 mm Hg on echocardiography, in separate models.

**Conclusion:**

Worsening in FVC and DLCO at 1 year, including a marginal decline in FVC ≥ 5% and DLCO ≥ 10%, is predictive of markedly reduced survival in fHP.

## Graphical abstract

A proportion of patients with fibrotic hypersensitivity pneumonitis have high mortality, and short‐term predictors of poorer survival are needed. Declines from baseline at 1 year in forced vital capacity (FVC) ≥ 10% and diffusion capacity of the lung for carbon monoxide (DLCO) ≥ 15%, but also marginal declines in FVC ≥ 5% and DLCO ≥ 10%, are predictive of increased mortality.

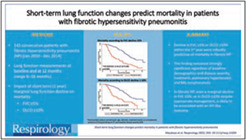

**See related** editorial

## INTRODUCTION

The majority of patients with hypersensitivity pneumonitis (HP) presenting to specialist centres have a chronic fibrotic form. Within the population of fibrotic HP (fHP) patients, a subgroup of patients display an accelerated rate of progression comparable to idiopathic pulmonary fibrosis (IPF).^1^, ^2^ The overlap with IPF is also evident in the often‐challenging differentiation of fHP from IPF in a multi‐disciplinary team (MDT) setting.^3^ The early identification of a rapidly progressive fibrosing lung disease is increasingly relevant in an era of anti‐fibrotic therapies, in which treatment guidelines are likely to increasingly broaden the spectrum of diagnoses for which anti‐fibrotic treatments are indicated.^4^

At the moment, known predictors of fHP mortality are the presence of traction bronchiectasis and honeycombing on thoracic computed tomography (CT),^1^, ^5^, ^6^, ^7^ lower baseline lung function,^5^, ^8^ lack of antigen identification/avoidance, and forced vital capacity (FVC) decline ≥ 10% within 6–12 months of follow‐up.^8^

The main aim of our study was to evaluate the impact of short‐term changes in FVC, including a marginal change of ≥5%, and in diffusion capacity of the lung for carbon monoxide (DLCO), including marginal change of ≥10%, on survival in patients with fHP.

## METHODS

### Patient selection and baseline characterization

Consecutive patients with a diagnosis of fHP presenting at the Royal Brompton Hospital (RBH) Interstitial Lung Disease (ILD) Unit from January 2010 to December 2014 were considered for this study. A diagnosis of fHP was made through MDT discussion, following integration of clinical, exposure history, CT, bronchoalveolar lavage (BAL) and histology data, when available, as per current guidelines.^9^

All‐cause mortality was collected for all patients until death, transplant, loss to follow‐up or end of the study period (30 March 2021).

Patients were included in this study if they had lung function measurements at first presentation to our unit (baseline), and at least one further lung function at 12 months (range: 6–18 months) from baseline. Patients who died within 12 months of baseline, without a follow‐up lung function test, were included in the baseline analysis but excluded from the lung function trends analysis. A categorical change in FVC was computed as a relative change of ≥5% or ≥10% from the absolute values at baseline, while a categorical change in DLCO was defined as a relative change of ≥10% or ≥15% from absolute values at baseline. Further details on clinical findings are available in Appendix S1 in the Supporting Information.

### Statistical analysis

Analyses were performed using STATA15.1 software (StataCorp, College Station, TX, USA). Determinants of mortality were assessed by Cox proportional hazards analysis. Covariates in multivariable analysis included demographic variables, the composite physiological index (CPI),^10^ used as a continuous variable to adjust for disease severity, presence of honeycombing on CT, and treatment (active treatment vs. no treatment, see Appendix S1 in the Supporting Information). The additional impact of BAL lymphocytosis and, in separate models, of raised pulmonary arterial systolic pressure (PASP) ≥ 40 mm Hg on echocardiography was assessed in separate multivariable models which included demographic variables and CPI. A *p*‐value of <0.05 was considered significant.

## RESULTS

### Baseline characteristics

Baseline characteristics of the 145 patients satisfying inclusion criteria for this study are summarized in Table 1. Briefly, mean age was 64.4 years (range: 33.0–87.2), 82 (56.6%) were female and 48 (33.1%) were ever smokers. Median baseline FVC was 67.5% (interquartile range [IQR]: 55.1–82.9) and DLCO was 37.4% (IQR: 29.5–47.2), while the median CPI was 54.7 (IQR: 43.9–60.6) (Table 1). FVC decline ≥ 5% in the first year was observed in 45 patients (31.0%), FVC ≥ 10% in 30 patients (20.7%), DLCO decline ≥ 10% in 43 (29.7%) and DLCO ≥ 15% in 35 (24.1%) patients. More than half of the cohort (83 [57.2%]) reported no obvious exposure history. The most frequently reported exposures included: avian (24 [16.6%]), mould or damp damage (18 [12.4%]) and feather bedding (2 [1.4%]). Of the 62 patients with reported exposures, all but two avoided further exposure to the potential antigen(s). Of the 141 patients tested for the presence of antibodies against common antigens, 46 (32.6%) showed positivity (Table S1 in the Supporting Information). One hundred and thirty (89.7%) patients were on active treatment (Table S2 in the Supporting Information). BAL at baseline was available for 100 (69.0%) patients. Histological confirmation of fHP was available for 36 (24.8%) patients. Diagnosis was reached by typical/compatible CT findings and evidence of exposure and/or BAL lymphocytosis ≥ 20% in 80 (55.2%) patients. In 29 (20%) patients, diagnosis was mainly based on CT findings, as BAL was not performed because of frailty/fHP severity, or patient choice. Echocardiogram within 12 months of baseline was available for 104 (71.7%) patients.

**TABLE 1:** Baseline characteristics of the study subjects

	*n* = 145
Age, years (range)	64.3 (33.0–87.2)
Gender, female (%)	82 (56.6)
Ethnicity, European (%)	76 (52.4)
Smoking status, never %)	97 (66.9)
Honeycombing on CT	19 (13.1)
Reported exposures^a^, *n* (%)	
Avian	24 (16.6)
Mould/damp	18 (12.4)
Feather bedding	2 (1.4)
Other	24 (16.6)
None	83 (57.2)
Baseline lung function	
DLCO % predicted	37.4 (29.5–47.2)
FVC % predicted	67.5 (55.1–82.9)
KCO % predicted	70.45 (60.3–86.3)
FEV1% predicted	68.2 (56.7–81.9)
CPI	54.7 (43.9–60.6)
BAL cellularity^b^	
Macrophages, %	55 (44–69)
Lymphocytes, %	23 (11.3–39)
Lymphocytes ≥ 20%	44 (44)
Lymphocytes ≥ 30%	32 (32)
Lymphocytes ≥ 40%	19 (19)
Neutrophils, %	6.3 (3.7–15.3)
Eosinophils, %	4 (1.7–6)
PASP ≥ 40 mm Hg^c^, *n* (%)	41 (39.4)

*Note*: Data are presented as mean (range) for age; all other data are presented as median (interquartile range) or number (percentage value) as appropriate.

Abbreviations: BAL, bronchoalveolar lavage; CPI, composite physiological index; CT, computed tomography; DLCO, diffusion capacity of the lung for carbon monoxide; FEV1, forced expiratory volume in 1 s; FVC, forced vital capacity; KCO, carbon monoxide transfer coefficient; PASP, pulmonary arterial systolic pressure.

^a^ Some patients had more than one reported exposure.

^b^ BAL was available for 100 patients.

^c^ Echocardiographic assessment within 12 months of baseline was available for 104 patients.

### Prognostic significance of baseline variables

There were 92 deaths (63.4%) during a median follow‐up of 4.8 years (range: 0.5–10.9). Baseline predictors of mortality included FVC % predicted (hazard ratio [HR]: 0.97, 95% CI: 0.96–0.98, *p* < 0.001), forced expiratory volume in 1 s (FEV1) % predicted (HR: 0.97, 95% CI: 0.96–0.98, *p* < 0.001), DLCO % predicted (HR: 0.97, 95% CI: 0.95–0.98, *p* < 0.001) and CPI (HR: 1.05, 95% CI: 1.03–1.07, *p* < 0.001). Age at baseline (HR: 1.04, 95% CI: 1.02–1.06, *p* = 0.002) and ethnicity (non‐Caucasian) (HR: 1.61, 95% CI: 1.07–2.44, *p* = 0.02) were also associated with mortality. The presence of honeycombing on CT, observed in 19 (13.1%) patients, was associated with mortality (HR: 2.13, 95% CI: 1.22–3.74, *p* = 0.008). In patients with an echocardiogram (*n* = 104), estimated PASP ≥ 40 mm Hg was predictive of mortality (HR: 1.92, 95% CI: 1.18–3.12, *p* = 0.008). In the 100 patients with BAL, lymphocyte percentage counts were associated with better survival, both when assessed as a continuous variable (HR: 0.96, 95% CI: 0.95–0.98, *p* < 0.001) and as different lymphocytosis cut‐offs (Table S3 in the Supporting Information). However, gender, smoking status, reported antigenic exposure or avoidance, and active treatment, were not observed to have prognostic value (Table S3 in the Supporting Information).

### Prognostic significance of lung function trends at 1 year

**FIGURE 1:**
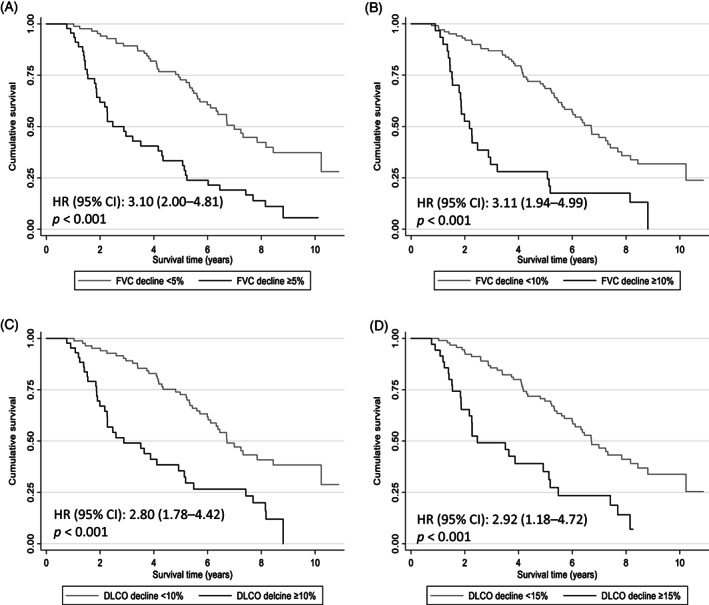
Survival according to decline in lung function at 1 year. Kaplan–Meier survival analysis grouped by decline at 1 year in (A) forced vital capacity (FVC) ≥ 5%, (B) FVC ≥ 10%, (C) diffusion capacity of the lung for carbon monoxide (DLCO) ≥ 10% and (D) DLCO ≥ 15%

On univariable analysis, a decline in FVC ≥ 10% at 1 year from baseline was highly predictive of mortality (HR: 3.11, 95% CI: 1.94–4.99, *p* < 0.001), with a median survival of 2.2 years compared to 6.7 years in those without FVC decline ≥ 10% (Figure 1B). Similarly, a decline in DLCO ≥ 15% at 1 year correlated with mortality (HR: 2.92, 95% CI: 1.18–4.72, *p* < 0.001), with a median survival of 2.5 years compared to 6.7 years in those without DLCO decline of ≥ 15% (Figure 1D). Even a marginal decline in FVC ≥ 5% was significantly predictive of mortality (HR: 3.10, 95% CI: 2.00–4.81, *p* < 0.001), with a median survival of 2.9 years compared to 7.0 years (Figure 1A). A marginal decline in DLCO ≥ 10% was also predictive of mortality (HR: 2.80 95% CI: 1.78–4.42, *p* < 0.001), with a median survival of 2.9 years compared to 6.7 years (Figure 1C).

All the above‐mentioned associations were confirmed as highly significant after adjusting for age, ethnicity, treatment (active or no treatment), presence of honeycombing on CT, and CPI (Table 2), and, in separate models, adjusting for alternative measure of ILD severity, baseline DLCO or FVC (Table S4 in the Supporting Information). The associations also remained significant when PASP ≥ 40 mm Hg was included in the multivariable analysis (Table S5 in the Supporting Information).

**TABLE 2:** Survival according to worsening in lung function at 1 year

	Univariable		Multivariable^a^	
	HR (95% CI)	*p*‐value	HR (95% CI)	*p*‐value
FVC decline ≥ 5%	3.10 (2.00–4.81)	<0.001	3.44 (2.12–5.59)	<0.001
FVC decline ≥ 10%	3.11 (1.94–4.99)	<0.001	3.31 (1.97–5.56)	<0.001
DLCO decline ≥ 10%	2.80 (1.78–4.42)	<0.001	3.45 (2.14–5.57)	<0.001
DLCO decline ≥ 15%	2.92 (1.18–4.72)	<0.001	3.61 (2.19–5.96)	<0.001

Abbreviations: CPI, composite physiological index; CT, computed tomography; DLCO, diffusion capacity of the lung for carbon monoxide; FVC, forced vital capacity; HR, hazard ratio.

^a^ Multivariable analysis correcting for age, ethnicity, treatment (active vs. no treatment as defined in Methods section), presence of honeycombing on CT and CPI.

To assess whether the link with worsening lung function was confounded by the presence of BAL lymphocytosis, we analysed the relationship between FVC decline (both ≥5% and ≥10%) and DLCO decline (≥10% and ≥15%), adjusting in separate analyses for different lymphocyte percentage cut‐offs (Table 3). The association with decline in FVC ≥ 5% and ≥10%, and with decline in DLCO ≥ 10% and ≥ 15% all remained highly significant, independent of the BAL lymphocyte thresholds of 20%, 30% and 40%, even after correcting for age, ethnicity, treatment, honeycombing on CT and CPI (Table 3).

**TABLE 3:** Survival according to change in lung function at 1 year, after adjusting for different thresholds of BAL lymphocytosis

	Multivariable^a^ lymphocytes ≥ 20%		Multivariable^a^ lymphocytes ≥ 30%		Multivariable^a^ lymphocytes ≥ 40%	
	HR (95% CI)	*p*‐value	HR (95% CI)	*p*‐value	HR (95% CI)	*p*‐value
FVC decline ≥ 5%	2.92 (1.63–5.21)	<0.001	2.70 (1.49–4.88)	0.001	2.69 (1.49–4.85)	0.001
FVC decline ≥ 10%	2.22 (1.15–4.30)	0.017	2.16 (1.13–4.11)	0.019	2.15 (1.15–4.05)	0.017
DLCO decline ≥ 10%	2.61 (1.47–4.62)	0.001	4.42 (1.36–4.30)	0.003	2.38 (1.34–4.23)	0.003
DLCO decline ≥ 15%	2.87 (1.54–5.33)	0.001	2.60 (1.34–5.01)	0.004	2.71 (1.46–5.04)	0.002

Abbreviations: BAL, bronchoalveolar lavage; CPI, composite physiological index; CT, computed tomography; DLCO, diffusion capacity of the lung for carbon monoxide; FVC, forced vital capacity; HR, hazard ratio.

^a^ Multivariable analysis correcting for age, ethnicity, honeycombing on CT, treatment (active vs. no treatment as defined in Methods), CPI and different BAL lymphocyte percentage cut‐offs.

We also performed a sensitivity analysis in which we excluded the 29 patients for whom diagnosis was mainly based on typical/compatible CT findings. In the remaining 116 patients, all four measures of lung function worsening remained significant on both univariable and multivariable analyses (Table S6 in the Supporting Information), including when PASP ≥ 40 mm Hg (Table S7 in the Supporting Information) and BAL lymphocyte thresholds (Table S8 in the Supporting Information) were included in separate multivariable analyses.

## DISCUSSION

In this study, we find that early lung function decline is predictive of an IPF‐like survival in patients with fHP, confirming the finding by Gimenez et al.^8^ that a decline in FVC ≥ 10% in the first year is an indicator of poor prognosis. In addition, we observe that even a marginal decline in FVC ≥ 5% within the first year is predictive of a markedly worse survival, with a median survival of only 2.9 years (HR: 3.10, 95% CI: 2.00–4.81, *p* < 0.001), highlighting the severity of outlook for this subgroup of patients. This is similar to the published findings in IPF, where a marginal decline (5%–10%) in FVC is associated with higher mortality.^11^ Furthermore, we find that a decline in DLCO within the first year also provides prognostic information, observing a similar impact on survival of a decline by ≥10% or ≥15%.

A decline in FVC approximating the 5% threshold is very close to the known SD for this measurement, safely stated at 5%, and may therefore represent either real progression of ILD or technical measurement variation.^12^ The higher the pre‐test probability of disease progression, the more likely it is that marginal decline reflects true deterioration. The fact that a threshold of 5% in FVC change is sufficient to predict survival suggests that in fHP the likelihood of progression is higher than in less progressive ILDs, where such a threshold is not predictive.^11^ That said, in individual cases, a change in FVC close to the 5% threshold could still represent measurement variation. However, our data suggest that, in fHP, even this marginal change should be taken seriously because of outcome significance, although corroboration with other variables suggestive of worsening could aid in determining whether change is real in the individual patient.

A median survival of only 26.0 months in patients with FVC decline of ≥10% within the first year is worse than the equivalent figure in the Gimenez et al.'s study (median survival of 53 months). This could be related to worse baseline severity in our cohort, although the absence of DLCO measurements in the Gimenez et al.'s study does not allow direct comparisons. Additionally, there were a greater proportion of patients without identifiable exposures in our cohort, a subgroup which is known to have a worse outcome.^13^ We did not however observe a difference in survival according to the history of exposure. The fact that a substantial percentage of patients with radiological and/or histological findings of fHP lack an identifiable exposure despite exhaustive investigation is well recognized.^9^ Indeed, some authors have suggested the term ‘cryptogenic HP’ to acknowledge this cohort, which may have many similarities with IPF. In this cohort, it was not possible to ascertain whether antigen avoidance allowed better survival than patients who did not subsequently avoid the identified exposure after diagnosis, as only two patients fell into the latter group. Finally, we cannot exclude the possibility that our results may have been confounded by the inclusion of IPF patients misclassified as fHP. However, the finding of an equally strong relationship between early lung function decline and mortality even after excluding the minority of patients in whom the diagnosis was mainly based on CT findings is reassuring and makes this possibility less likely.

A third of our cohort had a positive smoking history, although only two out of 48 were current smokers at their baseline visit. Although there is thought to be an inverse relationship between smoking history and development of HP,^14^ similar, or even greater, proportions (up to 57.9%) of HP patients with a positive smoking history have been reported.^15^, ^16^, ^17^, ^18^ Furthermore, the protective effect of smoking may not be applicable to ex‐smokers.^19^, ^20^

The majority of patients in this cohort were treated with corticosteroids and/or immunosuppressants. As ours was a retrospective study, it is not possible to accurately assess response to treatment. However, there was clearly a large proportion of patients with stable lung function at 1 year on corticosteroids and/or immunosuppressive treatment, a finding that was associated with a significantly better survival. Immunosuppression has been associated with stabilization of lung function in fHP,^21^, ^22^ although corticosteroid treatment was not associated with a survival benefit in a cohort of fHP patients.^23^ It is possible that anti‐inflammatory/immunosuppressive treatment has an adverse effect on a proportion of fHP patients, including those with shorter telomeres, while having a positive impact on survival in those with normal telomere lengths, as suggested by Adegunsoye et al.^24^ This possibility would require confirmation with a prospective study, as telomere studies were not available in this cohort. Early identification of fHP patients likely to have poor responses to immunosuppression is crucial, particularly now that anti‐fibrotic treatments have been shown to reduce FVC decline in patients with a progressive fibrotic phenotype regardless of the ILD entity.^25^, ^26^

One of the strengths of this study is the high proportion of patients with BAL at baseline. BAL lymphocytosis has been reported as a potential marker of a better outcome in patients with fHP. BAL lymphocytosis was associated with stabilization in FVC in response to immunosuppression,^27^ while De Sadeleer et al. reported better survival in patients with BAL lymphocytosis > 20%, with a marginal impact on early response of FVC following corticosteroid treatment.^28^ In this cohort, we confirm the association between BAL lymphocytosis and better outcomes in fHP. However, the association with early lung function change remained highly significant regardless of the BAL lymphocytosis thresholds analysed, suggesting an independent powerful impact of early lung function changes on outcome.

This study does have some limitations, mainly due to its retrospective nature. An echocardiogram within 12 months of baseline was not available for all patients. However, short‐term lung function worsening remained predictive of poor survival even after adjustment for PASP echocardiographic measurements available within a year from baseline. Although a detailed exposure history is routinely investigated in all new ILD patients referred to our unit, we do not employ a standardized written questionnaire, nor do we have availability of environmental hygienists to probe potential exposures in the patient's home or work environment. This could have missed potential exposures, as questionnaires and home/work visits may be better at identifying possible HP exposures than clinical history or specific IgG serologies.^29^ However, a number of large studies on HP have reported an identified exposure in less than 50% of the patients, a similar proportion to this cohort.^16^, ^30^, ^31^ Also, all‐cause mortality rather than respiratory cause‐only mortality was used, although this is a widely used outcome.^1^, ^7^, ^8^

In summary, in this cohort of consecutive patients with fHP, even a marginal decline in FVC by ≥5% and/or a marginal decline in DLCO by ≥10% is associated with a major increase in risk of death. In light of the very poor survival in a substantial subgroup of patients with fHP, there is a clear need to develop biomarkers of response to treatment (immunosuppressive) without having to wait for worsening. Furthermore, studies exploring the earliest time point from baseline at which change in lung function parameters is associated with survival are needed. There is a clear unmet need to identify this subgroup of patients with fHP much earlier in the course of their illness, by performing large well‐designed prospective studies to evaluate the role of early lung function decline in treatment decisions.

## CONFLICT OF INTEREST

None declared.

## AUTHOR CONTRIBUTION

**Caudio Macaluso:** Data curation (equal); formal analysis (equal); investigation (equal); methodology (equal); writing – review and editing (equal). **Cristina Boccabella:** Data curation (equal); formal analysis (equal); investigation (equal); methodology (equal); writing – review and editing (equal). **Maria Kokosi:** Data curation (equal); investigation (equal); writing – review and editing (equal). **Nishanth Sivarasan:** Data curation (equal); investigation (equal); writing – review and editing (equal). **Vasilis Kouranos:** Data curation (equal); investigation (equal); writing – review and editing (equal). **Peter M. George:** Data curation (equal); investigation (equal); writing – review and editing (equal). **George Margaritopoulos:** Data curation (equal); investigation (equal); writing – review and editing (equal). **Philip L. Molyneaux:** Data curation (equal); investigation (equal); writing – review and editing (equal). **Felix Chua:** Data curation (equal); investigation (equal); writing – review and editing (equal). **Toby M. Maher:** Data curation (equal); investigation (equal); writing – review and editing (equal). **Gisli R. Jenkins:** Data curation (equal); investigation (equal); writing – review and editing (equal). **Andrew G. Nicholson:** Investigation (equal); writing – review and editing (equal). **Sujal R. Desai:** Conceptualization (equal); data curation (equal); investigation (equal); writing – review and editing (equal). **Anand Devaraj:** Conceptualization (equal); data curation (equal); investigation (equal); writing – review and editing (equal). **Athol U. Wells:** Conceptualization (equal); methodology (equal); writing – review and editing (equal). **Elisabetta A. Renzoni:** Conceptualization (equal); data curation (equal); formal analysis (equal); investigation (equal); methodology (equal); writing – original draft (equal); writing – review and editing (equal). **Carmel J.W. Stock:** Data curation (equal); formal analysis (equal); investigation (equal); methodology (equal); writing – original draft (equal); writing – review and editing (equal).

## HUMAN ETHICS APPROVAL DECLARATION

This study was performed in accordance with the Declaration of Helsinki. This human study was approved by Royal Brompton and Harefield Hospitals (approval: IRAS: 236745). Adult participant consent was not required as the research was based on retrospective review of previously collected non‐identifiable information, and patient consent was not required for institutional approval.

## Supporting information

Supporting information

**Visual Abstract** Short‐term lung function changes predict mortality in patients with fibrotic hypersensitivity pneumonitis.

## Data Availability

The data that support the findings of this study are available from the corresponding author upon reasonable request.
