# Supplementary material for: Short‐term lung function changes predict mortality in patients with fibrotic hypersensitivity pneumonitis
Source: Respirology. 2022 Jan 12;27(3):202–8. doi: 10.1111/resp.14204 (PMC9302621; doi:10.1111/resp.14204)
Supplement: Supplementary file 1 — Supporting information [file RESP-27-202-s002.docx]

**SUPPORTING INFORMATION**

**Short term lung function changes predict mortality in patients with fibrotic hypersensitivity pneumonitis**

Claudio Macaluso^1,2^, Cristina Boccabella^1,3^, Maria Kokosi^1^, Nishanth Sivarasan^4^,

Vasilis Kouranos^1^, Peter M George^1,5^, George Margaritopoulos^1,6,7^, Philip L Molyneaux^1,5^, Felix Chua^1^, Toby M Maher^1,5,8^, Gisli R Jenkins^1^, Andrew G Nicholson^5,9^, Sujal Desai^4^, Anand Devaraj^4^, Athol U Wells^1,5^, Elisabetta A Renzoni*^1,5^, Carmel JW Stock*^1,5^

^1^ Interstitial Lung Disease Unit, Royal Brompton and Harefield Clinical Group, Guy’s and St Thomas’ NHS Foundation Trust, London, UK

^2^ INRCA/IRCCS, "L.Mandic" Hospital Merate (LC) - Department of Pneumology, Merate, Italy

^3^ Department of Medical and Surgical Sciences, Fondazione Policlinico Universitario "A. Gemelli" – IRCCS, University of the Sacred Heart, Rome, Italy.

^4^ Department of Radiology, Royal Brompton and Harefield Clinical Group, Guy’s and St Thomas’ NHS Foundation Trust, London, UK

^5^ Margaret Turner Warwick Centre for Fibrosing Lung Disease, National Heart and Lung Institute, Imperial College London, London, UK

^6^ ILD Unit, London North West University Hospital Healthcare Trust, London, UK

^7^ Faculty of Biology, Medicine and Health, The University of Manchester, Manchester, UK

^8^ Hastings Centre for Pulmonary Research and Division of Pulmonary, Critical Care and Sleep Medicine, Keck School of Medicine, University of Southern California, Los Angeles, USA

^9^Department of Histopathology, Royal Brompton and Harefield Clinical Group, Guy’s and St Thomas’ NHS Foundation Trust, London, UK

*contributed equally

**Appendix S1-METHODS**

**Patient selection and baseline characterisation**

All ILD patients assessed at our Unit undergo a standardised set of questions on exposures encountered at work, home, and related to hobbies. The presence of pulmonary hypertension (PH) on echocardiography, within 12 months of baseline, was defined as a pulmonary arterial systolic pressure (PASP) of ≥40 mm Hg.^1^ Smoking history was defined as never or ever (former or current). Treatment was defined as corticosteroid (prednisolone/methylprednisolone ≥1mg/day) and/or immunosuppressant (azathioprine, cyclophosphamide, hydroxychloroquine, methotrexate, mycophenolate mofetil) therapy (Supplementary Table S1). Treatment status was sub-categorised as ‘active treatment’ (treatment instituted within 3 months of baseline visit or continuation of pre-existing treatment) or ‘no treatment’ (no therapy within 3 months of follow-up from baseline visit).

**Lung function tests**

All lung function tests were performed in the same lung function laboratory at RBH, including FVC, forced expiratory volume in 1 second (FEV1), and DLCO levels, as previously reported.^2^ The composite physiologic index (CPI), a functional index of lung fibrosis severity, was calculated as follows: CPI = 91.0 - (0.65 x DLCO % predicted) - (0.53 x FVC % predicted) + (0.34 x FEV1 % predicted).^3^ Although originally developed for IPF patients, it has subsequently been used in other ILDs, and found to have good validity as a prognostic marker.^2, 4-6^

**Bronchoalveolar lavage (BAL)**

BAL samples were prepared based on international guidelines.^7^ Samples were taken at bronchoscopy with fluid collected in polypropylene bottles and then sent to the pathology department. Fluid was then centrifuged and cytospins subsequently stained with a May-Grunwald-Giemsa (MGG) stain and 300 cells counted manually, with percentages of inflammatory cells recorded.

**CT assessment of honeycombing**

All CT scans were evaluated by a Radiologist with expertise in ILD imaging (NS) for the presence of honeycombing, defined as closely approximated well-defined subpleural ring-shadows or cystic air spaces.^8^ We chose to include CT honeycombing in the survival analysis, as previously reported to be significantly associated with survival in fHP patients.^9^

**References**

^1^ Plastiras, S. C., Karadimitrakis, S. P., Kampolis, C., Moutsopoulos, H. M. and Tzelepis, G. E. Determinants of pulmonary arterial hypertension in scleroderma. *Semin Arthritis Rheum*. 2007; **6**: 392-6.

^2^ Jacob, J., Bartholmai, B. J., Egashira, R., Brun, A. L., Rajagopalan, S., Karwoski, R., Kokosi, M., Hansell, D. M. and Wells, A. U. Chronic hypersensitivity pneumonitis: identification of key prognostic determinants using automated CT analysis. *BMC Pulm Med*. 2017; **1**: 81.

^3^ Wells, A. U., Desai, S. R., Rubens, M. B., Goh, N. S., Cramer, D., Nicholson, A. G., Colby, T. V., du Bois, R. M. and Hansell, D. M. Idiopathic pulmonary fibrosis: a composite physiologic index derived from disease extent observed by computed tomography. *Am J Respir Crit Care Med*. 2003; **7**: 962-9.

^4^ Stock, C. J. W., Hoyles, R. K., Daccord, C., Kokosi, M., Visca, D., De Lauretis, A., Alfieri, V., Kouranos, V., Margaritopoulos, G., George, P. M., Molyneaux, P. L., Chua, F., Maher, T. M., Abraham, D. J., Ong, V., Donovan, J., Sestini, P., Denton, C. P., Wells, A. U. and Renzoni, E. A. Serum markers of pulmonary epithelial damage in systemic sclerosis-associated interstitial lung disease and disease progression. *Respirology*. 2021; **5**: 461-468.

^5^ Walsh, S. L., Wells, A. U., Sverzellati, N., Keir, G. J., Calandriello, L., Antoniou, K. M., Copley, S. J., Devaraj, A., Maher, T. M., Renzoni, E., Nicholson, A. G. and Hansell, D. M. An integrated clinicoradiological staging system for pulmonary sarcoidosis: a case-cohort study. *Lancet Respir Med*. 2014; **2**: 123-30.

^6^ Meier, C., Freiburghaus, K., Bovet, C., Schniering, J., Allanore, Y., Distler, O., Nakas, C. and Maurer, B. Serum metabolites as biomarkers in systemic sclerosis-associated interstitial lung disease. *Sci Rep*. 2020; **1**: 21912.

^7^ Meyer, K. C., Raghu, G., Baughman, R. P., Brown, K. K., Costabel, U., du Bois, R. M., Drent, M., Haslam, P. L., Kim, D. S., Nagai, S., Rottoli, P., Saltini, C., Selman, M., Strange, C., Wood, B. and American Thoracic Society Committee on, B. A. L. i. I. L. D. An official American Thoracic Society clinical practice guideline: the clinical utility of bronchoalveolar lavage cellular analysis in interstitial lung disease. *Am J Respir Crit Care Med*. 2012; **9**: 1004-14.

^8^ Hansell, D. M., Bankier, A. A., MacMahon, H., McLoud, T. C., Muller, N. L. and Remy, J. Fleischner Society: glossary of terms for thoracic imaging. *Radiology*. 2008; **3**: 697-722.

^9^ Salisbury, M. L., Gu, T., Murray, S., Gross, B. H., Chughtai, A., Sayyouh, M., Kazerooni, E. A., Myers, J. L., Lagstein, A., Konopka, K. E., Belloli, E. A., Sheth, J. S., White, E. S., Holtze, C., Martinez, F. J. and Flaherty, K. R. Hypersensitivity Pneumonitis: Radiologic Phenotypes Are Associated With Distinct Survival Time and Pulmonary Function Trajectory. *Chest*. 2019; **4**: 699-711.

**Table S1-Antibody positivity for common antigens**

| **Antibody positivity** | **Patients n=141** |
| --- | --- |
| *Aspergillus* | 41 (29.1%) |
| Avian* | 8 (6.3%) |
| *Micropolispora foeni*^‡^ | 4 (3.2%) |

* 127 patients tested, ^‡^ 125 patients tested

**Table S2-Type of treatment at baseline**

| **Type of treatment at baseline *** | **Patients n=145** |
| --- | --- |
| None | 15 (10.3%) |
| Corticosteroids only | 74 (51.0%) |
| Corticosteroids + conventional immunosuppressants^‡^ | 56 (38.6%) |

* Treatment at baseline was considered the treatment instituted within 3 months since first visit or continuation of pre-existing treatment

^‡^ Azathioprine, cyclophosphamide, hydroxychloroquine, methotrexate, mycophenolate mofetil

**Table S3- Prognostic significance of baseline variables**

|  | **HR (95% CI)** | **p value** |
| --- | --- | --- |
| **Age** | 1.04 (1.02-1.06) | 0.002 |
| **Gender** | 0.96 (0.63-1.45) | 0.84 |
| **Ethnicity** | 1.61 (1.07-2.44) | 0.023 |
| **Smoking status** | 1.16 (0.76-1.78) | 0.49 |
| **Antigen Exposure** | 1.22 (0.81-1.84) | 0.35 |
| **Antigen avoidance** | 1.23 (0.81-1.88) | 0.33 |
| **Autoantibody positivity** | 0.93 (0.59-1.47) | 0.76 |
| **Lymphocyte count** | 0.96 (0.95-0.98) | <0.001 |
| **Lymphocytes ≥20%** | 0.54 (0.32-0.90) | 0.018 |
| **Lymphocytes ≥30%** | 0.38 (0.22-0.67) | 0.001 |
| **Lymphocytes ≥40%** | 0.34 (0.16-0.76) | 0.008 |
| **Neutrophil count** | 1.02 (0.99-1.05) | 0.11 |
| **Eosinophil count** | 1.00 (0.96-1.04) | 0.99 |
| **Monocyte count** | 0.86 (0.55-1.35) | 0.51 |
| **PASP ≥40mm Hg on echo** | 1.92 (1.18-3.12) | 0.008 |
| **Honeycombing on CT** | 2.13 (1.22-3.74) | 0.008 |
| **Treatment** | 1.14 (0.59-2.21) | 0.69 |
| **FVC** | 0.97 (0.96-0.98) | <0.001 |
| **FEV1** | 0.97 (0.96-0.98) | <0.001 |
| **DLCO** | 0.97 (0.95-0.98) | <0.001 |
| **KCO** | 1.01 (0.99-1.02) | 0.09 |
| **CPI** | 1.05 (1.03-1.07) | <0.001 |

PASP: pulmonary arterial systolic pressure, CT: computed tomography, FVC: forced vital capacity, FEV1: forced expiratory volume in 1 second, DLCO: diffusing capacity for carbon monoxide, KCO: Carbon monoxide transfer coefficient, CPI: composite physiologic index

**Table S4- Survival according to change in lung function at one year- correcting for baseline lung function**

|  | **DLCO** | | **FVC** | |
| --- | --- | --- | --- | --- |
|  | **HR (95% CI)** | **p value** | **HR (95% CI)** | **p value** |
| **Decline FVC ≥5%** | 3.41 (2.11-5.50) | <0.001 | 3.71 (2.32-5.96) | <0.001 |
| **Decline FVC ≥10%** | 3.34 (2.00-5.58) | <0.001 | 3.59 (2.15-5.98) | <0.001 |
| **Decline DLCO ≥10%** | 3.53 (2.19-5.70) | <0.001 | 3.10 (1.92-5.02) | <0.001 |
| **Decline DLCO ≥15%** | 3.80 (2.30-6.28) | <0.001 | 3.10 (1.87-5.11) | <0.001 |

Correcting for baseline severity as well as age, ethnicity, presence of honeycombing on CT and treatment (active vs no treatment).DLCO: diffusing capacity of the lung for carbon monoxide, FVC: forced vital capacity.

**Table S5- Survival according to change in lung function at one year-correcting for PASP**

|  | **CPI** | | **DLCO** | | | **FVC** |  |
| --- | --- | --- | --- | --- | --- | --- | --- |
|  | **HR (95% CI)** | **p value** | **HR (95% CI)** | **p value** | | **HR (95% CI)** | **p value** |
| **Decline FVC ≥5%** | 2.79 (1.60-4.85) | <0.001 | 2.75 (1.59-4.74) | <0.001 | 2.80 (1.62-4.82) | | <0.001 |
| **Decline FVC ≥10%** | 2.89 (1.53-5.46) | 0.001 | 2.88 (1.52-5.45) | 0.001 | 3.00 (1.59-5.63) | | 0.001 |
| **Decline DLCO ≥10%** | 3.35 (1.92-5.82) | <0.001 | 3.41 (1.96-5.92) | <0.001 | 3.16 (1.81-5.50) | | <0.001 |
| **Decline DLCO ≥15%** | 3.33 (1.87-5.94) | <0.001 | 3.50 (1.96-6.26) | <0.001 | 2.97 (1.67-5.28) | | <0.001 |

Correcting for baseline severity as well as age, ethnicity, presence of honeycombing on CT, treatment (active vs no treatment), and PASP ≥40mm Hg on echocardiography. CPI: composite physiological index, DLCO: diffusing capacity of the lung for carbon monoxide, FVC: forced vital capacity, PASP: pulmonary arterial systolic pressure.

**Table S6- Survival according to change in lung function at one year- correcting for baseline lung function, excluding patients in whom diagnosis was mainly based on CT findings**

|  | **Univariable** | | **Multivariable*** | | | | | |
| --- | --- | --- | --- | --- | --- | --- | --- | --- |
|  |  |  | **CPI** | | **DLCO** | | **FVC** | |
|  | **HR (95% CI)** | **p value** | **HR (95% CI)** | **p value** | **HR (95% CI)** | **p value** | **HR (95% CI)** | **p value** |
| **Decline FVC ≥5%** | 3.10 (1.87-5.12) | <0.001 | 3.78 (2.14-6.66) | <0.001 | 3.64 (2.08-6.35 | <0.001 | 4.07 (2.30-7.19) | <0.001 |
| **Decline FVC ≥10%** | 3.66 (2.13-6.28) | <0.001 | 4.55 (2.41-8.59) | <0.001 | 4.51 (2.40-8.46) | <0.001 | 4.45 (2.35-8.43) | <0.001 |
| **Decline DLCO ≥10%** | 2.82 (1.67-4.74) | <0.001 | 3.33 (1.93-5.74) | <0.001 | 3.47 (2.01-5.99) | <0.001 | 2.84 (1.64-4.94) | <0.001 |
| **Decline DLCO ≥15%** | 2.85 (1.63-4.97) | <0.001 | 3.50 (1.96-6.26) | <0.001 | 3.80 (2.12-6.83) | <0.001 | 2.79 (1.55-5.03) | 0.001 |

*Correcting for baseline severity as well as age, ethnicity, presence of honeycombing on CT and treatment (active vs no treatment). CPI: composite physiological index, DLCO: diffusing capacity of the lung for carbon monoxide, FVC: forced vital capacity.

**Table S7- Survival according to change in lung function at one year- correcting for PASP, excluding patients in whom diagnosis was mainly based on CT findings**

|  | **CPI** | | **DLCO** | | | **FVC** | |
| --- | --- | --- | --- | --- | --- | --- | --- |
|  | **HR (95% CI)** | **p value** | **HR (95% CI)** | **p value** | | **HR (95% CI)** | **p value** |
| **Decline FVC ≥5%** | 2.90 (1.53-5.50) | 0.001 | 2.78 (1.48-5.21) | 0.001 | 2.96 (1.56-5.61) | | 0.001 |
| **Decline FVC ≥10%** | 3.55 (1.66-7.62) | 0.001 | 3.52 (1.64-7.54) | 0.001 | 3.73 (1.74-8.01) | | 0.001 |
| **Decline DLCO ≥10%** | 3.20 (1.68-6.11) | <0.001 | 3.32 (1.74-6.35) | <0.001 | 2.84 (1.49-5.43) | | 0.002 |
| **Decline DLCO ≥15%** | 2.94 (1.50-5.77) | 0.002 | 3.17 (1.61-6.22) | 0.001 | 2.43 (1.23-4.81) | | 0.01 |

Correcting for baseline severity as well as age, ethnicity, presence of honeycombing on CT, treatment (active vs no treatment), and PASP ≥40mm Hg on echocardiography. FVC: forced vital capacity, DLCO: diffusing capacity of the lung for carbon monoxide, PASP: pulmonary arterial systolic pressure.

**Table S8 - Survival according to change in lung function at one year- adjusting for different thresholds of BAL lymphocytosis, excluding patients in whom diagnosis was mainly based on CT findings**

|  | **Multivariable***  **Lymphocytes ≥20%** | | **Multivariable***  **Lymphocytes ≥30%** | | **Multivariable***  **Lymphocytes ≥40%** | |
| --- | --- | --- | --- | --- | --- | --- |
|  | **HR (95% CI)** | **p value** | **HR (95% CI)** | **p value** | **HR (95% CI)** | **p value** |
| **Decline FVC ≥5%** | 4.52 (2.18-9.38) | <0.001 | 4.05 (1.93-8.48) | <0.001 | 4.04 (1.88-8.68) | <0.001 |
| **Decline FVC ≥10%** | 3.93 (1.71-8.96) | 0.001 | 4.08 (1.81-9.22) | 0.001 | 3.67 (1.65-8.19) | 0.001 |
| **Decline DLCO ≥10%** | 2.95 (1.52-5.72) | 0.001 | 2.66 (1.39-5.11) | 0.003 | 2.62 (1.36-5.05) | 0.004 |
| **Decline DLCO ≥15%** | 3.55 (1.73-7.27) | 0.001 | 3.10 (1.44-6.68) | 0.004 | 3.19 (1.56-6.52) | 0.002 |

*Multivariable analysis correcting for age, ethnicity, honeycombing on CT, treatment (active vs no treatment as defined in methods), CPI, and different BAL lymphocyte percentage cut-offs. FVC: forced vital capacity, DLCO: diffusing capacity of the lung for carbon monoxide.
