# Supplementary material for: Short‐term lung function changes predict mortality in patients with fibrotic hypersensitivity pneumonitis
Source: Respirology. 2022 Jan 12;27(3):202–8. doi: 10.1111/resp.14204 (PMC9302621; doi:10.1111/resp.14204)
Supplement: Supplementary file 2 — Visual Abstract Short‐term lung function changes predict mortality in patients with fibrotic hypersensitivity pneumonitis. [file RESP-27-202-s001.pdf]

Short-term lung function changes predict mortality in patients with fibrotic hypersensitivity pneumonitis

METHODS

- 145 consecutive patients with fibrotic hypersensitivity pneumonitis (HP) (Jan 2010 - Dec 2014)
- Lung function measurements at baseline and at 12 months (range 6–18 months)
- Impact of short term (1 year) marginal lung function decline on mortality
  - FVC ≥5%
  - DLCO ≥10%

RESULTS

Mortality according to FVC decline ≥5%

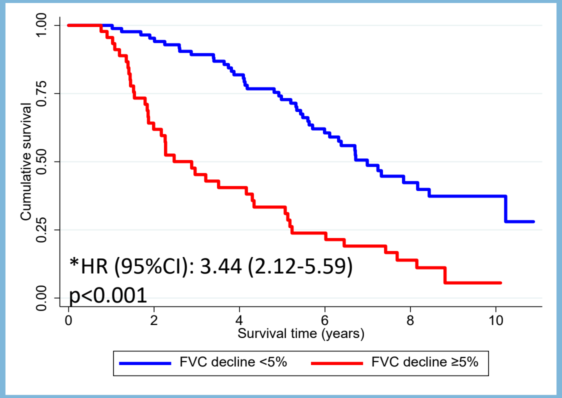

Mortality according to DLCO decline ≥ 10%

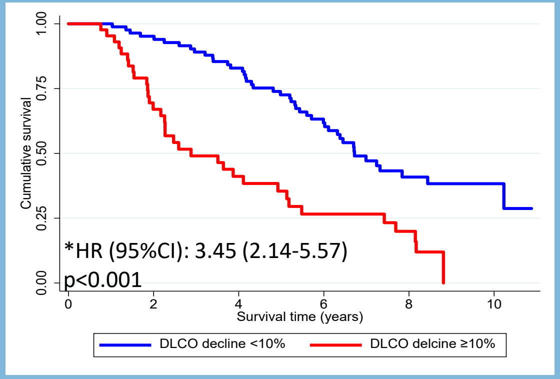

*Multivariable analysis correcting for age, ethnicity, treatment (active vs no treatment) presence of honeycombing on CT, and baseline disease severity (CPI)

SUMMARY

- Decline in FVC ≥5% or DLCO ≥10% within the 1st year were robustly predictive of mortality in fibrotic HP
- This finding remained strongly significant regardless of baseline demographics and disease severity, treatment, pulmonary hypertension and BAL lymphocytosis
- In fibrotic HP, even a marginal decline in FVC ≥5% or in DLCO ≥10% despite appropriate management, is likely to be associated with an IPF-like outcome

Respirology

Short-term lung function changes predict mortality in patients with fibrotic hypersensitivity pneumonitis

Macaluso et al. Respirology 2022; DOI: 10.1111/resp.14204
